# Does Lung Ultrasound Have a Role in the Clinical Management of Pregnant Women with SARS COV2 Infection?

**DOI:** 10.3390/ijerph18052762

**Published:** 2021-03-09

**Authors:** Maria Grazia Porpora, Lucia Merlino, Luisa Masciullo, Rossella D’Alisa, Gabriella Brandolino, Cecilia Galli, Casimiro De Luca, Francesco Pecorini, Giovanni Battista Fonsi, Andrea Mingoli, Cristiana Franchi, Alessandra Oliva, Lucia Manganaro, Claudio Maria Mastroianni, Maria Grazia Piccioni

**Affiliations:** 1 Department of Maternal and Child Health and Urological Sciences, Sapienza, University of Rome, Policlinico Umberto I, Viale del Policlinico 155, 00161 Rome, Italy; mariagrazia.porpora@uniroma1.it (M.G.P.); lucia.merlino@uniroma1.it (L.M.); luisa.masciullo@uniroma1.it (L.M.); rossella.dalisa@uniroma1.it (R.D.); gabriella.brandolino@uniroma1.it (G.B.); deluca.1637044@studenti.uniroma1.it (C.D.L.); francesco.pecorini@uniroma1.it (F.P.); mariagrazia.piccioni@uniroma1.it (M.G.P.); 2 Department of Surgery, “Pietro Valdoni”, Sapienza University of Rome, Policlinico Umberto I, Viale del Policlinico 155, 00161 Rome, Italy; battista.fonsi@hotmail.com (G.B.F.); andrea.mingoli@uniroma1.it (A.M.); 3 Department of Public Health and Infectious Diseases, Sapienza University of Rome, Policlinico Umberto I, Viale del Policlinico 155, 00161 Rome, Italy; cristiana.franchi@uniroma1.it (C.F.); alessandra.oliva@uniroma1.it (A.O.); claudio.mastroianni@uniroma1.it (C.M.M.); 4 Department of Radiological, Oncological, and Pathological Sciences, Sapienza, University of Rome, Policlinico Umberto I, Viale del Policlinico 155, 00161 Rome, Italy; lucia.manganaro@uniroma1.it

**Keywords:** SARS-CoV-2 infection, pregnancy, lung ultrasound (LUS) examination, chest computed tomography

## Abstract

Severe Acute Respiratory Syndrome coronavirus 2 (SARS-CoV-2) infection is a major health threat. Pregnancy can lead to an increased susceptibility to viral infections. Although chest computed tomography (CT) represents the gold standard for the diagnosis of SARS-CoV-2 pneumonia, lung ultrasound (LUS) could be a valid alternative in pregnancy. The objectives of this prospective study were to assess the role of LUS in the diagnosis of lung involvement and in helping the physicians in the management of affected patients. Thirty pregnant women with SARS-CoV-2 infection were admitted at the obstetrical ward of our Hospital. Mean age was 31.2 years, mean gestational age 33.8 weeks. Several LUS were performed during hospitalization. The management of the patients was decided according to the LUS score and the clinical conditions. Mean gestational age at delivery was at 37.7 weeks, preterm birth was induced in 20% of cases for a worsening of the clinical conditions. No neonatal complications occurred. In 9 cases with a high LUS score, a chest CT was performed after delivery. CT confirmed the results of LUS, showing a significant positive correlation between the two techniques. LUS seems a safe alternative to CT in pregnancy and may help in the management of these patients.

## 1. Introduction

Severe Acute Respiratory Syndrome coronavirus 2 (SARS-CoV-2) infection has rapidly spread throughout and on 30 January 2020 the World Health Organization (WHO) declared a public health emergency of international interest (PHEIC) [1]. Nowadays, at least 90 million SARS-CoV-2 positive cases have been confirmed worldwide [2].

The rising mortality rate of SARS-CoV-2 has highlighted the need to identify the most susceptible populations. Pregnant women seem to be at high risk of SARS COV 2 infection, even if only a few studies have investigated maternal and neonatal outcomes [3,4]. 

Pregnant women are known to be highly susceptible to severe forms and complications of infections by previous coronaviruses, and from the beginning of SARS-CoV-2 pandemia, they have been considered as a vulnerable group [5,6]. 

In 2020, as part of SARS-CoV-2 surveillance, the US Centers for Disease Control and Prevention (CDC) received the reports of 1,300,938 women of reproductive age (15–44 years) who had given positive test results for SARS-CoV-2. Among the symptomatic women, approximately 5.7% of them were pregnant and more likely to be admitted to an intensive care unit (ICU) and to receive invasive ventilation. This suggests that pregnancy may increase the risk of a worse evolution of the SARS-CoV-2 infection [7].

Pregnancy is a peculiar condition characterized by several changes that involve not only the anatomy and the physiology but also the immune system, which may influence the response to a viral infection. During pregnancy there is a reduction of cellular immune response in favor of humoral responses, mediated by a shift in CD4+ T cell population Th2/Th1 towards Th2 phenotype [8]. However, the presence of high levels of progesterone (a hormone with immunomodulatory properties) may have a role in protecting lungs after a viral infection, stimulating reparation processes [9,10,11]. 

In addition to the immunological changes, several anatomical and physiological modifications occur in the respiratory system during pregnancy, resulting in a reduced ability to compensate for any lung disease [12,13]. Furthermore, it is well known that pregnancy is a predisposing condition for hypercoagulability; indeed, there is an increase in the majority of clotting factors, a decrease in the natural anticoagulants’ levels and in the fibrinolytic activity, aimed at limiting the maternal bleeding during delivery and placental expulsion [14,15,16]. This prothrombotic state, as well as the increased intra-vascular inflammation, exacerbated in case of infection, may expose pregnant women with SARS-CoV-2 infection to an increased thromboembolic risk [17].

The cardiorespiratory changes in pregnancy may also cause a delay in the diagnosis due to the possible presence of gestational rhinitis which may mask the coryzal symptoms of SARS-CoV-2 infection [18]. The most common symptoms in both pregnant and non-pregnant women with SARS-CoV-2 infection are fever, cough and myalgia, but pregnant women are more likely to show an asymptomatic course than non-pregnant ones [19].

The gold standard for the diagnosis of SARS-CoV-2 is the viral nucleic acid detection in naso-pharyngeal swabs, sputum and bronchoalveolar secretions by (real-time) reverse transcription polymerase chain reaction (RT-PCR) [20]. 

Nowadays, chest computed tomography (CT) represents the best imaging method for screening, preclinical diagnosis of interstitial pneumonia and evaluation of severity as well as the most sensitive method for diagnosis of SARS-CoV-2 infection [21,22]. According to the current evidence, typical lung changes at imaging appear earlier than clinical symptoms; in addition, recent studies addressed the importance of Chest CT in patients with typical symptoms of SARS-CoV-2 and false negative RT-PCR results [21,23,24]. Therefore, lung imaging plays a crucial role in early diagnosis of SARS-CoV-2 pneumonia.

Unfortunately, CT has some limitations: it requires patient mobilization and it exposes the patients to ionizing radiations, which restricts its use during pregnancy. In pregnancy a radiation-free diagnostic tool, such as lung ultrasound (LUS), may provide an alternative imaging technique, for the diagnosis of lung involvement until delivery [25]. 

Typical LUS patterns suggestive of SARS-CoV-2 pneumonia have been described, [26] providing a useful guide for assessing the lungs involvement in pregnant women [27]. Buonsenso et al. recently reported that LUS has a higher sensitivity than chest X-ray in the diagnosis of SARS-CoV-2 pneumonia in these patients [28].

The application of standardized scanning methods assign a score to each pulmonary area and then to calculate the degree of pulmonary ventilation by summing up each area (the higher the score, the lower the ventilation) [29]. 

Few investigations have been conducted so far on the diagnostic accuracy of LUS, however it may have an important role in quantifying the degree of pulmonary engagement in pregnant patients with a positive nasopharyngeal swab [30]. 

The primary objective of this study is to assess the role of LUS in detecting the degree of ventilation dysfunction and performing a constant monitoring of lung function in pregnant women affected by SARS-CoV-2 disease. The secondary objective is to analyze whether LUS may guide the medical team to define the most appropriate therapeutic strategy and improve pregnancy outcomes. 

## 2. Materials and Methods

### 2.1. Study Population 

We conducted a prospective observational study from 1 March 2020 to 30 November 2020, on symptomatic pregnant women, admitted to the Covid 19- Obstetrical ward of the Department of Maternal and Child Health of the Policlinico Umberto I Hospital, Sapienza University of Rome, after having undergone a nasopharyngeal swab for the detection of SARS-CoV-2 disease by (rRT-PCR) testing, which confirmed a SARS-CoV-2 infection.

### 2.2. Inclusion Criteria

- Singleton pregnancy
- A positive result to the nasopharyngeal swab and detection of SARS-CoV-2 by RT-PCR testing
- Age between 18 and 45 years
- A body mass index (BMI) ≤ 30
- Absence of previous or current pregnancy comorbidities
- Absence of maternal comorbidities

### 2.3. Exclusion Criteria

- Twin pregnancies
- A previous SARS-CoV-2 infection
- A maternal age >45 years
- BMI > 30
- A presence of maternal or pregnancy-related comorbidities.

Nasopharyngeal swab samples were collected for the screening of all patients admitted to the Hospital and SARS-CoV-2 RNA was detected by using real time RT-PCR assay (RealStar SARS-CoV2 RT-PCR, Altona Diagnostics, Hamburg, Germany). Following logistical and clinical needs, other molecular methods were used (GeneFinder COVID-19 Plus RealAmp Kit, Elitech, Anyang-si, Gyeonggi-do, South Korea; DiaSorin Molecular Simplexa COVID-19 Direct EUA assay, DiaSorin Molecular, Cypress, California, U.S.A.; Xpert Xpress SARS-CoV-2 assay, Cepheid, Sunnyvale, California, USA). All tests and procedures were performed following the manufacturers’ protocols. A positive PCR Test was considered diagnostic for a SARS COV 2 infection.

Maternal age, body mass index (BMI: weight/height^2^), parity, and gestational age were recorded at the admission in the hospital. All symptoms suggestive of SARS-CoV-2 infection such as fever, cough, myalgia, general weakness and /or impaired olfactory-gustatory sensitivity were also recorded. 

Definition of pneumonia or severe pneumonia was based on the WHO interim guidance and included clinical symptoms of pneumonia (fever, cough, dyspnea, fast breathing) with or without signs of severe pneumonia such as respiratory rate > 30 breaths/min, severe respiratory distress, or SpO2 < 90% on room air [31]. 

Laboratory routine analysis including C-reactive protein (CRP), procalcitonin (PCT), and D-dimer, peripheral blood oxygen saturation were daily performed in all patients and, when necessary, hemogasanalysis was undertaken. 

**Figure 1:**
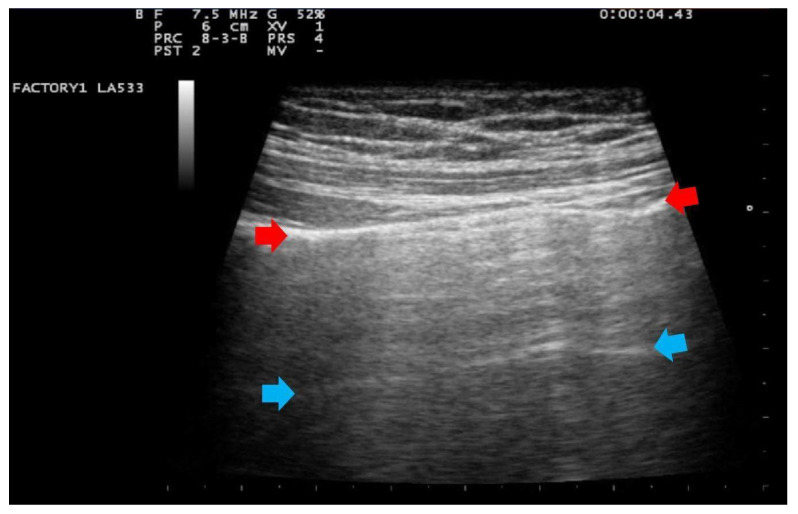
Lung US images obtained with linear transducers., frequency 7.5 MHz, focus on the pleural line. Trapezoidal view. The pleura line (indicated by red arrows) is continuous. Below, horizontal artifacts (A line), indicated by blue arrows, may be visible. This pattern is classified as score 0.

**Figure 2:**
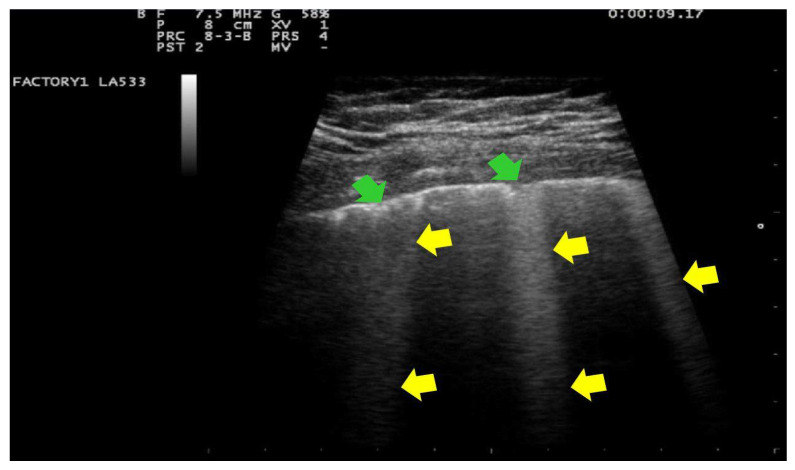
Lung US images obtained with linear transducers, frequency 7.5 MHz, focus on the pleural line. Trapezoidal view. The pleural line is not continuous. Below the point of discontinuity (indicated by green arrows), vertical areas of white (or B lines) are visible (indicated by yellow arrows). B-lines occupying <50% the screen. This pattern is classified as score 1p.

**Figure 3:**
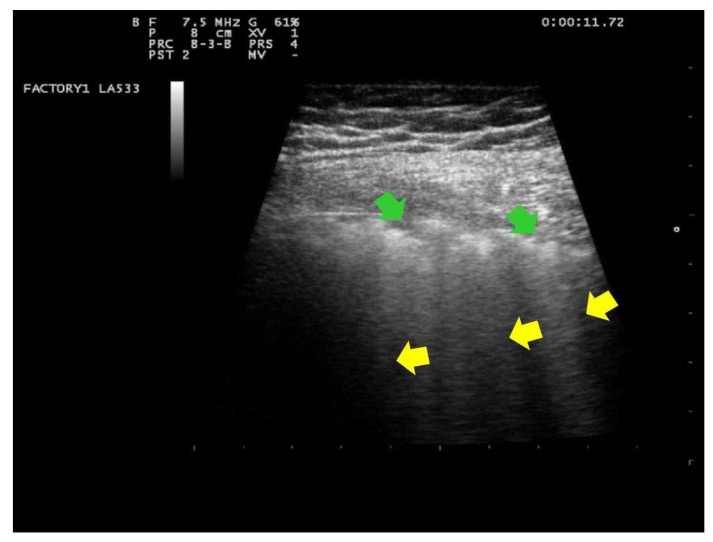
Lung US images obtained with linear transducers, frequency 7.5 MHz, focus on the pleural line. Trapezoidal view. The pleural line is not continuous. Below the point of discontinuity (indicated by green arrows), vertical areas of white (or B lines) are visible (indicated by yellow arrows). B-lines occupying >50% the screen. This pattern is classified as score 2.

**Figure 4:**
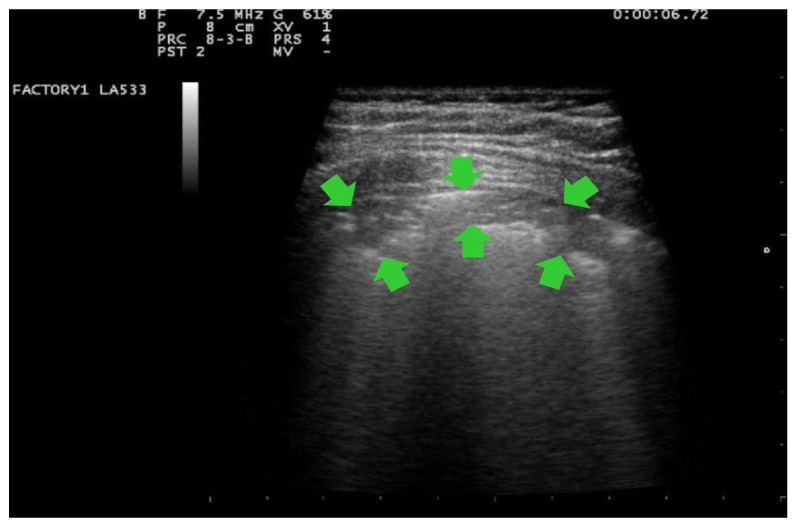
LUS image of the patient with extensive pneumonia obtained with linear transducers, frequency 7.5 MHz, focus on the pleural line. Trapezoidal view. Presence of large consolidations (at least >1 cm) (indicated by green arrows). This pattern is classified as score 3.

In addition, in all cases, at the patient’s admission and the day after delivery, a clinician with 15 years of experience (G.B.F.) performed a LUS evaluation with a portable ultrasound device (MyLab 25 Gold, Esaote SpA, Genoa, Italy), using convex and/or linear probes that transmit ultrasounds at frequencies of 2.5 and 7.5 MHz, respectively. Linear probe was used for evaluating lung involvement with the details of the pleural line and subpleural space; while the convex probe was used to assess the presence of pericardial effusion. The LUS investigation was conducted according to the 12-zone method, both in the supine and lateral positions [29]. Intercostal scans were performed in the three different areas of each hemithorax (anterior, lateral and posterior) and the superior and inferior segments. A total of six specific regions for each lung (right and left) were analyzed for 60 s. The presence of the following parameters was assessed on pulmonary ultrasound investigation: (1) an irregular thickened pleural line; (2) A-lines; (3) B-lines (>= 3, confluent or nonconfluent); (4) consolidations; (5) B-lines and consolidation location of upper, middle or inferior lobes; (6) multilobe involvement; (7) bilateral distribution; (8) air bronchogram sign; (9) pleural effusion; (10) pericardial effusion. For each patient, the degree of pulmonary involvement was calculated using the score proposed by Bouhemad et al. [29] and adopted by other authors [32,33]. The lung involvement was scored as follows:

Score 0: predominant A-lines or <3 separated B-lines. (Figure 1)
Score 1: Score 1: at least three B-lines or confluent B-lines which occupy ≤ 50% of the screen without irregularities of pleural line. (Figure 2) Score 1p: B-lines with a clearly irregular pleural line.
Score 2: confluent B-lines which occupy >50% of the screen without irregularities pleural line. Score 2p: confluent B-lines with a clearly irregular pleural line (Figure 3).
Score 3: consolidations of large dimensions (at least >1 cm). (Figure 4)

Signs of pleural effusion should be reported if present.

The final score was obtained by adding the scores of each area, ranging from 0 to 36. This score provides an estimate of the lung aeration, the higher the score the lower is the lung aeration. The association between LUS and the presence of clinical symptoms allowed to classify the disease in mild, moderate and severe for LUS-scores ≤ 6, between 7and 20 and >20, respectively. 

According to the SARS-CoV-2 institutional therapeutic protocol and together with dedicated Infectious Diseases consultants, the patients with the most severe degree of infection, based on clinical symptoms (dyspnea requiring supplemental oxygen therapy) and/or a LUS score > 7, received medical therapy with intravenous corticosteroids (dexamethasone 6 mg every 24 h), enoxaparin (either as a prophylactic or therapeutic dosages, when appropriate) and oral azithromycin 500 mg die for 7 days, the latter in case of suspicion of bacterial superinfections. All patients underwent clinical and obstetrical ultrasound investigations and daily cardiotocographic exams in order to monitor the pregnancy and the fetal wellbeing. Gestational age at birth, type of delivery, neonatal Apgar score and neonatal weight were also recorded.

After delivery, the patients with high LUS score and/or clinical persistence of respiratory symptoms underwent a chest CT to evaluate the presence of interstitial lung consolidation. Radiological terms as ground glass opacity (GGO), crazy-paving pattern and pulmonary consolidation have been defined on the standard glossary for Thoracic imaging reported by the Fleischner Society [34]. The CT scan performed to assess the pulmonary involvement was scored according to a CT-based semi-quantitative score for each lobe proposed by Pan et al. as follow: 0, no involvement; 1, <5% involvement; 2, 5–25% involvement; 3, 26–50% involvement; 4, 51–75% involvement; 5, >75% involvement. The global CT results from the sum of each lobar score varying from 0 to 25 [35]. The study was approved by the Ethics Committee of Policlinico Umberto I, Sapienza University of Rome (Protocol number 109/2020). All patients provided written informed consent and procedures followed were in accordance with the Helsinki declaration of 1975, as revised in 2000. 

### 2.4. Statistics

Analysis was performed using the Statistical Product and Service Solutions software (SPSS) version 20 for Windows (SPSS Inc., Chicago, IL, USA). Descriptive analyses were presented as frequency with percentage, mean and standard deviation for all variables considered. The Shapiro-Wilk test was performed to test for a normal distribution. As the variables did not have a normal distribution the Kruskall-Wallis and the Mann-Whitney non parametric tests were carried out; we also analyzed variables’ correlations using Pearson and Rho Spearman Correlation tests; confidence interval was 95% and a *p*-value < 0.05 was considered as statistically significant. 

## 3. Results

Thirty pregnant women with a confirmed infection of SARS-CoV-2 were included in the study. The main patients’ characteristics are reported in Table 1.

The median age of the patients was 33.5 years and the median BMI 27. These patients presented different combinations of clinical symptoms; the most common were fever (26.7%), cough (36%), dyspnea (6.7%) and general weakness (33%).

All patients showed high blood levels of CRP and PCT. The median value of D-dimer was 2320 µg/mL. The mean oxygen peripheral saturation was 97% (±1.8). Only in one patient, with the highest LUS score, the oxygen saturation dropped to 93% and she required oxygen supplementation. During the hospitalization 22 patients (73%) had a LUS score ≤6, eight patients (27%) had a score ≥7, showing a clinical picture of a moderate or severe disease; among these, three (10%) reached a score ≥20. These eight patients received medical treatment with corticosteroids, enoxaparin at the prophylactic dosage and azithromycin according to the therapeutic protocol of our hospital. Only one patient with severe respiratory symptoms received enoxaparin at therapeutic dosage and remdesivir (loading dose 200 mg 1st day followed by 100 mg/die for a total of 5 days of therapy) immediately after delivery. No adverse reactions were observed during remdesivir administration. The remaining 22 patients received medical treatment with enoxaparin at prophylactic dosage. The characteristics of different clinical symptoms, length of hospitalization and LUS score are reported on Table 2.

A worsening of the clinical condition led to a preterm delivery induction (<37 weeks of gestation, the lowest gestational age was 32 weeks) in six patients (20% of cases), after the administration of corticosteroids (betamethasone 12 mg intramuscular injection every 24 h, for a total of 2 doses) to achieve fetal lung maturation. Overall, the median gestational age at delivery was at 38 weeks; 12 patients (40%) underwent cesarean section; 18 patients (60%) had a vaginal delivery. The mean neonatal Apgar index at 1 min was 9 (±0.50) and at 5 min was 9.5 (±0.50), the mean neonatal weight was 3341.80 gr (±311.3). No neonatal complications occurred and no SARS-CoV-2 infections were found in the newborns.

After delivery, nine patients, with a high LUS score and/or persistent clinical symptoms, underwent chest CT which showed the presence of interstitial pneumonia in all cases with a median CT score of 4 (from a minimum of 1 and a maximum of 16). (Table 3).

**Figure 5:**
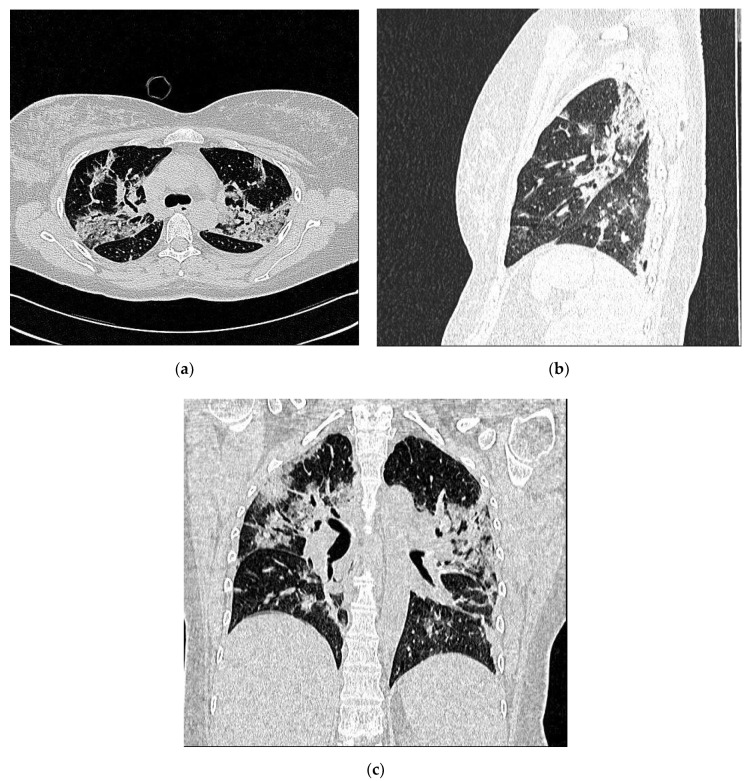
CHEST CT shows extensive interstitial pneumonia on the axial plane (**a**) with crazy paving pattern and consolidation area. Multiplanar reconstruction (MPR) on coronal and sagittal planes (**b**,**c**) demonstrate scissure thickening and fibrosis.

The patient with severe respiratory symptoms had CT images of an extensive interstitial pneumonia with a score of 16. Her LUS and chest CT findings are shown in Figure 4 and Figure 5.

The statistical analysis of our results showed a significant positive correlation between the gestational age at birth and the mode of delivery (*p* < 0.05) and between the LUS score and CT score (*p* < 0.01), while there was a significant negative correlation between the LUS score and the gestational age at birth (*p* < 0.01) and SpO_2_ (*p* < 0.01) (Table 4).

No statistically significant correlation was found between the mode of delivery (vaginal delivery or cesarean section) and the severity of lung involvement and clinical characteristics.

The three patients with high CT and high LUS scores were admitted to the infectious disease ward. They were discharged after a mean of 17.6 (±2.5) days. Before resigning from the hospital, the patients underwent LUS examination again. The exams showed a normal lung’s picture and the absence of micro-consolidations were observed in all cases. Indeed, their score was 0 in all lobes (predominant A-lines or three separated B-lines). All women will be followed-up and undergo LUS 6 months after the discharge.

## 4. Discussion

SARS-CoV-2 infection represents a global health emergency which is still challenging health providers and clinical researchers all over the world. Therefore, it is crucial to avoid the potential devastating effects, through new diagnostic tools and clinical evidence [36,37,38].

Pregnant women seem to be predisposed to severe SARS-CoV-2 infections due to the immune, physiological and anatomical changes occurring during this period [39,40].

Recent studies have analyzed the course of the infection during pregnancy, showing its polyhedral clinical manifestations, ranging from an asymptomatic condition to an acute respiratory distress syndrome (ARDS) [7,41,42,43,44]. The effect of pregnancy on the SARS-CoV-2 infection is still unclear. A recent study, using CT to detect the severity of lung involvement, showed that pregnancy does not worsen the course of SARS-CoV-2 pneumonia [45]. In fact, several studies mainly reported mild respiratory symptoms during pregnancy [46,47,48] and they often reported a normal course of the gestation with the absence of maternal and/or fetal complications or differences in pregnancy outcome compared to the general population [44,49,50,51,52,53,54,55,56,57,58,59,60,61].

On the other hand, other studies have found that SARS-CoV-2 infection during pregnancy could compromise its course, being frequently associated with a worse obstetric outcome if compared with unaffected women at the same gestational age [62,63]. According to recent scientific evidence, it seems that the presence of previous maternal risk factors; such as an age > 35 years, a BMI > 30, a chronic hypertension or a pre-existing diabetes could be responsible of a worse clinical course, leading to a higher rate of admission in intensity care unit (ICU) [19,64,65]. In our research we have excluded patients with a BMI > 30 and/or age > 45 years because we wanted to reduce the risk of other gestational comorbidities, like diabetes, hypertension and pre-eclampsia which could worsen the clinical course of the infection. In our series, almost all had mild symptoms. Severe symptoms were observed only in a 44 years-old woman with a BMI of 29, without any other anamnestic risk factors, with a clinical and a radiological picture of a severe interstitial pneumonia (a LUS score of 23 and a CT score of 16). She was treated with the therapeutic protocol for SARS-CoV-2 pneumonia of our hospital, including remdesivir immediately after delivery. Remdesivir was chosen because it has been recently approved by FDA as a promising and safe antiviral drug against SARS-CoV2 in patients with severe respiratory symptoms; indeed, there are some preliminary reports showing that it could be safe and well tolerated also in pregnancy [65].

Among the techniques that allow to understand the grade of severity of this disease, CT represents the widely accepted gold standard to evaluate the stage of pulmonary disease [66]. The most frequent CT imaging pattern is characterized by typical pulmonary paintings (bilateral ground-glass opacity with crazy-paving patterns that worsen as infection progresses). Francone et al. have proposed a Chest CT score, based on the extent of lobar involvement, which seems to well correlate with the inflammatory status and disease severity; this could be helpful to assist medical staff to timely establish symptomatic treatment. Indeed, they observed a strong correlation between the disease severity and semi quantitative CT score. In particular, for patients with a score ≥ 18 an increased mortality was reported [24]. Recent studies showed the importance of CT to point out a diagnosis of interstitial pneumonia especially in asymptomatic patients [24,35,67,68].

Therefore, it is reasonable to consider chest imaging as a key step in order to achieve a complete clinical evaluation of patients, even if the use of chest CT scan in pregnancy should be restricted to severe respiratory impairment.

LUS imaging is hand-held, bed-side, rapid, reproducible, easy to learn and cost-saving method, compared with other imaging techniques. It has been recently proposed by Chinese Critical Care Ultrasound Study Group and Italian Academy of Thoracic Ultrasound as an essential tool to show pulmonary patterns that are frequently associated with this infection [26,27,28]. This technique avoids the exposure to ionizing radiation and it can be easily performed in the hospital room. Therefore, it could be a valid alternative tool in pregnant women also because it can safely be repeated to monitor the lung involvement, even daily, until delivery [69,70]. In our study we investigated if LUS could be used for screening and early detection of lung involvement in pregnant women with a confirmed diagnosis of SARS-CoV-2 infection. It was performed in all patients at hospital admission as a primary imaging assessment, to detect a pulmonary involvement, and it was repeated during the patients’ hospitalization. In our experience LUS showed to be a safe and accurate diagnostic procedure that allowed us to monitor the lung involvement of the infection. These findings are in line with those recently reported by Buonsenso et al. [28].

Chest CT, which was performed after delivery in patients with high LUS score or persistent respiratory symptoms, showed a score up to 4 with an involvement <10% in five cases, a score between 5 to 8 with an involvement of 10–25% in three women, while in the patient with a LUS score of 23 CT showed the 60–70% of lung involvement with a score of 16. Even if the CT findings were more precise in defining the pulmonary impairment, our results could suggest a possible correlation between LUS and CT. Our data are in agreement with the results of previous studies which compared LUS and chest CT in patients with SARS-CoV-2 respiratory impairment [71]. We also evaluated its possible role in the clinical and therapeutic decision-making process, which was the secondary objective of our study. Falgarone et al. recently showed that LUS may have a role in the assessments of oxygen requirements in affected non-pregnant patients [72] and, at the same way, the management of our patients was chosen on the basis of the clinical course and the LUS findings; the cases with a score >7 received a medical treatment for SARS-CoV-2 infection. In our study, the worsening of clinical conditions in 6 patients led to an iatrogenic preterm delivery as soon as fetal lung maturity was achieved after prophylactic therapy with betamethasone.

Our approach in the management of pregnant patients with SARS-CoV-2 infection is in line with two recent studies in which the authors reported that the patient’s clinical symptoms and LUS score can guide the medical team to perform the most appropriate management of patients and improve pregnancy outcomes [28,73].

A recent study has reported a high risk of spontaneous preterm delivery in cases of SARS-CoV-2 infection in pregnancy [74]; this condition is often associated with obstetric complications like Small for Gestational Age (SGA) newborns, preterm delivery and a high rate of cesarean section. Despite the increasing risk of preterm birth, due to the inflammatory status, spontaneous preterm birth did not occur in our cohort; in fact, the majority of our women (80%) had a term delivery. These findings are in line with other studies which reported that preterm births are more frequently induced by obstetricians who take care of the patients, while a spontaneous preterm delivery caused by the disease has been rarely observed [56,64,75].

Although several studies found a high rate of neonatal intensive care unit (NICU) admission [76,77,78] in our series we did not detect neonatal complications or respiratory problems even in the preterm newborns. In addition, none of the newborns had a positive swab for SARS-CoV-2 infection. These findings confirm what reported by some studies that have questioned the hypothesis of a possible vertical transmission of the virus during pregnancy and delivery [79].

Our results suggest that LUS score could rapidly define the clinical status of pregnant women and, thanks to its safety, it can be repeated several times without maternal and/or fetal risks. In fact, even if the amount of radiation used in a normal CT scan, probably does not harm the fetus after a single standard scan, [80] it could not be repeated several times in pregnancy as may be required in some cases of SARS-CoV-2 symptomatic infection. LUS can be considered an emerging technique which allows a quick screening in infected patients and a useful tool in pregnancy [81]. It has been reported that this technique has a sensitivity of 95% and a specificity of 90%, [82,83,84] being a reliable tool in pregnancy.

According to our results and literature findings, the management of each case could be planned on the basis of the clinical status and the pulmonary involvement observed at LUS [85]. We are aware that our study has some limitations. Firstly, the number of patients is too small for a correct analysis and interpretation of the results; secondly, the follow up of these patients is still ongoing. However, despite these limitations, our data strongly support the use of LUS in this group of patients.

## 5. Conclusions

LUS represents an emerging and cost saving technique. This is useful for screening, early detection and follow up of the pulmonary involvement in pregnant women with SARS-CoV-2 infection. It also seems to play an important role in the decision-making process. We have observed a positive correlation between LUS score and CT score carried out after delivery, which strengthens the reliability of LUS as an alternative diagnostic method to CT in pregnant women. In our patients LUS is proven to be safe, reliable, sensitive, easily repeatable, and could be a guide to define the most appropriate strategy and to improve clinical and pregnancy outcomes. Although LUS could not replace CT, which is the gold standard for lung evaluation, LUS may be considered a reliable tool in pregnancy. However, further prospective studies are required to confirm our data and the benefits of this imaging method in pregnant women with SARS-CoV-2 infection [28,86,87,88].

## Figures and Tables

**Table 1:** Descriptive analysis of main patients’ characteristics using median and (interquartile range) or number (%).

PATIENT CHARACTERISTICS (N = 30)	DESCRIPTIVE ANALYSIS Median (Range)
AGE (years)	33.5 (27–35)
BMI	27 (23–29)
GESTATIONAL AGE AT TEST POSITIVITY (weeks)	36 (28–38)
PARTIAL SATURATION OF OXYGEN (SpO2)%)	97.5 (96–99)
D-DIMER VALUE (µg/mL)	2320 (2070–3489)
LUS SCORE	3 (0–8)
CT SCORE	4 (2–7.5)
GESTATIONAL AGE AT DELIVERY (weeks)	38 (37–39)
MODE OF DELIVERY Vaginal Delivery Cesarean Section	N. % 18 (60) 12 (40)

**Table 2:** Characteristics of patients’ symptoms, length of hospitalization according to LUS score.

LUS SCORE (N)	NO CLINICAL SYMPTOMS N (%)	COUGH N (%)	FEVER N (%)	WEAKNESS N (%)	DYSPNOEA N (%)	LENGTH of HOSPITALIZATION Mean (SD)
≤ 6 (22)	14 (63.6)	3 (13.6)	2 (9.1)	5 (22.7)	0	6.7 (±2.3)
>7 and <20 (5)	0	5 (100)	3 (60)	2 (40)	0	10 (±2)
≥20 (3)	0	3 (100)	3 (100)	3 (100)	2 (66.7)	17.6 (±2.5)

**Table 3:** LUS and CT scores in the 9 women who underwent both examinations.

Patient ID	LUS SCORE	TC SCORE
1	23	16
2	20	7
3	10	3
4	10	3
5	12	5
6	10	4
7	22	8
8	8	1
9	6	1

**Table 4:** Correlation between LUS score, CT score, mode of delivery (vaginal or cesarean delivery), gestational age (GA), and partial saturation of oxygen (SpO_2_).

**LUS SCORE**	**CORRELATIONS**	**Coefficient**	***p*-Value**
	SpO_2_	−0.499	<0.01
	Mode of delivery	0.225	0.81
	GA at birth	−0.652	<0.01
	CT score	0.888	<0.01

## Data Availability

The data presented in this study are available on request from the corresponding author. The data are not publicly available due to our privacy policy.

## References

[1] Cheruiyot I., Henry B.M., Lippi G. (2020). Is there evidence of intra-uterine vertical transmission potential of COVID-19 infection in samples tested by quantitative RT-PCR?. Eur. J. Obstet. Gynecol. Reprod. Biol..

[2] Dong E., Du H., Gardner L. (2020). An interactive web-based dashboard to track COVID-19 in real time. Lancet Infect. Dis..

[3] Zaigham M., Andersson O. (2020). Maternal and perinatal outcomes with COVID-19: A systematic review of 108 pregnancies. Acta Obstet. Gynecol. Scand..

[4] Bellos I., Pandita A., Panza R. (2021). Maternal and perinatal outcomes in pregnant women infected by SARS-CoV-2: A meta-analysis. Eur. J. Obstet. Gynecol. Reprod. Biol..

[5] Di Mascio D., Khalil A., Saccone G., Rizzo G., Buca D., Liberati M., Vecchiet J., Nappi L., Scambia G., Berghella V. (2020). Outcome of coronavirus spectrum infections (SARS, MERS, COVID-19) during pregnancy: A systematic review and meta-analysis. Am. J. Obstet. Gynecol. MFM.

[6] Wong S.F., Chow K.M., Leung T.N., Ng W.F., Ng T.K., Shek C.C., Ng P.C., Lam P.W., Ho L.C., To W.W. (2004). Pregnancy and perinatal outcomes of women with severe acute respiratory syndrome. Am. J. Obstet. Gynecol..

[7] Zambrano L.D., Ellington S., Strid P., Galang R.R., Oduyebo T., Tong V.T., Woodworth K.R., Nahabedian J.F., Baumgartner A.E., Gilboa S.M. (2020). Update: Characteristics of Symptomatic Women of Reproductive Age with Laboratory-Confirmed SARS-CoV-2 Infection by Pregnancy Status—United States, 22 January–3 October. MMWR. Morb. Mortal. Wkly. Rep..

[8] Piccinni M.P., Romagnani S. (1996). Regulation of fetal allograft survival by hormone-controlled Th1- and Th2-type cytokines. Immunol. Res..

[9] Siiteri P.K., Febres F., Clemens L.E., Chang R.J., Gondos B., Stites D. (1977). Progesterone and Maintenance of Pregnancy: Is Progesterone Nature’s Immunosuppressant?. Ann. N. Y. Acad. Sci..

[10] Druckmann R., Druckmann M.A. (2005). Progesterone and the immunology of pregnancy. J. Steroid Biochem. Mol. Biol..

[11] Hall O.J., Klein S.L. (2017). Progesterone-based compounds affect immune responses and susceptibility to infections at diverse mucosal sites. Mucosal Immunol..

[12] Ramsey P.S., Ramin K.D. (2001). Pneumonia in Pregnancy. Obstet. Gynecol. Clin. N. Am..

[13] Cruikshank D.P., Wigton T.R., Hays P.M., Gabbe S.G., Niebyl J.R., Simpson J.L. (1996). Maternal physiology in pregnancy. Obstetrics: Normal and Problem Pregnancies.

[14] Bremme A.K. (2003). Haemostatic changes in pregnancy. Best Pract. Res. Clin. Haematol..

[15] Dahlman T., Hellgren M., Blombäck M. (1985). Changes in Blood Coagulation and Fibrinolysis in the Normal Puerperium. Gynecol. Obstet. Investig..

[16] Riordan O.M.N., Higgins J.R. (2003). Haemostasis in normal and abnormal pregnancy. Best Pract. Res. Clin. Obstet. Gynaecol..

[17] Di Renzo G.C., Giardina I. (2020). Coronavirus disease 2019 in pregnancy: Consider thromboembolic disorders and thromboprophylaxis. Am. J. Obstet. Gynecol..

[18] Dashraath P., Wong J.L.J., Lim M.X.K., Lim L.M., Li S., Biswas A., Choolani M., Mattar C., Su L.L. (2020). Coronavirus disease 2019 (COVID-19) pandemic and pregnancy. Am. J. Obstet. Gynecol..

[19] Jafari M., Pormohammad A., Neshin S.A.S., Ghorbani S., Bose D., Alimohammadi S., Basirjafari S., Mohammadi M., Ivey R.C., Razizadeh M.H. (2021). Clinical characteristics and outcomes of pregnant women with COVID-19 and comparison with control patients: A systematic review and meta-analysis. Rev. Med. Virol..

[20] Zhao J.Y., Yan J.Y., Qu J.M. (2020). Interpretations of “Diagnosis and Treatment Protocol for Novel Coronavirus Pneumonia (Trial Version 7)”. Chin. Med. J..

[21] Fang Y., Zhang H., Xie J., Lin M., Ying L., Pang P., Ji W. (2020). Sensitivity of Chest CT for COVID-19: Comparison to RT-PCR. Radiology.

[22] Zu Z.Y., Di Jiang M., Xu P.P., Chen W., Ni Q.Q., Lu G.M., Zhang L.J. (2020). Coronavirus Disease 2019 (COVID-19): A Perspective from China. Radiology.

[23] Bernheim A., Mei X., Huang M., Yang Y., Fayad Z.A., Zhang N., Diao K., Lin B., Zhu X., Li K. (2020). Chest CT Findings in Coronavirus Disease-19 (COVID-19): Relationship to Duration of Infection. Radiology.

[24] Francone M., Iafrate F., Masci G.M., Coco S., Cilia F., Manganaro L., Panebianco V., Andreoli C., Colaiacomo M.C., Zingaropoli M.A. (2020). Chest CT score in COVID-19 patients: Correlation with disease severity and short-term prognosis. Eur. Radiol..

[25] Gargani L., Picano E. (2015). The risk of cumulative radiation exposure in chest imaging and the advantage of bedside ultrasound. Crit. Ultrasound J..

[26] Peng Q.Y., Wang X.T., Zhang L.N. (2020). Chinese Critical Care Ultrasound Study Group (CCUSG). Findings of lung ultrasonography of novel corona virus pneumonia during the 2019–2020 epidemic. Intensiv. Care Med..

[27] Moro F., Buonsenso D., Moruzzi M.C., Inchingolo R., Smargiassi A., Demi L., Larici A.R., Scambia G., Lanzone A., Testa A.C. (2020). How to perform lung ultrasound in pregnant women with suspected COVID. Ultrasound Obstet. Gynecol..

[28] Buonsenso D., Raffaelli F., Tamburrini E., Biasucci D.G., Salvi S., Smargiassi A., Inchingolo R., Scambia G., Lanzone A., Testa A.C. (2020). Clinical role of lung ultrasound for diagnosis and monitoring of COVID-19 pneumonia in pregnant women. Ultrasound Obstet. Gynecol..

[29] Bouhemad B., Mongodi S., Via G., Rouquette I. (2015). Ultrasound for “Lung Monitoring” of Ventilated Patients. Anesthesiology.

[30] Youssef A., Cavalera M., Azzarone C., Serra C., Brunelli E., Casadio P., Pilu G. (2020). The use of lung ultrasound during the COVID-19 pandemic. J. Popul. Ther. Clin. Pharmacol..

[31] World Health Organization Clinical Management of COVID-19: Interim Guidance. https://apps.who.int/iris/handle/10665/332196.

[32] Gargani L., Aboumarie S.H., Volpicelli G., Corradi F., Pastore M.C., Cameli M. (2020). Why, when, and how to use lung ultrasound during the COVID-19 pandemic: Enthusiasm and caution. Eur. Hear. J. Cardiovasc. Imaging.

[33] Fonsi G.B., Sapienza P., Brachini G., Andreoli C., De Cicco M.L., Cirillo B., Meneghini S., Pugliese F., Crocetti D., Fiori E. (2020). Is Lung Ultrasound Imaging a Worthwhile Procedure for Severe Acute Respiratory Syndrome Coronavirus 2 Pneumonia Detection?. J. Ultrasound Med..

[34] Hansell D.M., Bankier A.A., MacMahon H., McLoud T.C., Müller N.L., Remy J. (2008). Fleischner Society: Glossary of Terms for Thoracic Imaging. Radiology.

[35] Pan F., Ye T., Sun P., Gui S., Liang B., Li L., Zheng D., Wang J., Hesketh R.L., Yang L. (2020). Time Course of Lung Changes at Chest CT during Recovery from Coronavirus Disease 2019 (COVID-19). Radiology.

[36] Casadio P., Youssef A., Arena A., Gamal N., Pilu G., Seracchioli R. (2020). Increased rate of ruptured ectopic pregnancy in COVID-19 pandemic: Analysis from the North of Italy. Ultrasound Obstet. Gynecol..

[37] Diaz A., Sarac B.A., Schoenbrunner A.R., Janis J.E., Pawlik T.M. (2020). Elective surgery in the time of COVID. Am. J. Surg..

[38] Iacobucci G. (2020). Covid-19: All non-urgent elective surgery is suspended for at least three months in England. BMJ.

[39] Mor G., Aldo P., Alvero A.B. (2017). The unique immunological and microbial aspects of pregnancy. Nat. Rev. Immunol..

[40] Liu H., Wang L.L., Zhao S.J., Kim K.J., Mor G., Liao A.H. (2020). Why are pregnant women susceptible to COVID-19? An immunological viewpoint. J. Reprod. Immunol..

[41] Lei D., Wang C., Li C., Fang C., Yang W., Chen B., Wei M., Xu X., Yang H., Wang S. (2020). Clinical characteristics of COVID-19 in pregnancy: Analysis of nine cases. Chin. J. Perinat. Med..

[42] Zhang L., Jiang Y., Wei M., Cheng B.H., Zhou X.C., Li J., Tian J.H., Dong L., Hu R.H. (2020). Analysis of the pregnancy out-comes in pregnant women with COVID-19 in Hubei Province. Zhonghua Fu Chan Ke Za Zhi.

[43] Huang W., Zhao Z., He Z., Liu S., Wu Q., Zhang X., Qiu X., Yuan H., Yang K., Tang X. (2020). Unfavorable outcomes in pregnant patients with COVID. J. Infect..

[44] Sutton D., Fuchs K., Alton D.M., Goffman D. (2020). Universal Screening for SARS-CoV-2 in Women Admitted for Delivery. N. Engl. J. Med..

[45] Liu H., Liu F., Li J., Zhang T., Wang D., Lan W. (2020). Clinical and CT imaging features of the COVID-19 pneumonia: Focus on pregnant women and children. J. Infect..

[46] Wu Z., McGoogan J.M. (2020). Characteristics of and Important Lessons from the Coronavirus Disease 2019 (COVID-19) Outbreak in China: Summary of a Report of 72 314 Cases from the Chinese Center for Disease Control and Prevention. JAMA.

[47] Xu L., Yang Q., Shi H., Lei S., Liu X., Zhu Y., Wu Q., Ding X., Tian Y., Hu Q. (2020). Clinical presentations and outcomes of SARS-CoV-2 infected pneumonia in pregnant women and health status of their neonates. Sci. Bull..

[48] Chen L., Li Q., Zheng D., Jiang H., Wei Y., Zou L., Feng L., Xiong G., Sun G., Wang H. (2020). Clinical Characteristics of Pregnant Women with Covid-19 in Wuhan, China. N. Engl. J. Med..

[49] Yang Z., Wang M., Zhu Z., Liu Y. (2020). Coronavirus disease 2019 (COVID-19) and pregnancy: A systematic review. J. Matern. Neonatal Med..

[50] Chen H., Guo J., Wang C., Luo F., Yu X., Zhang W., Li J., Zhao D., Xu D., Gong Q. (2020). Clinical characteristics and intrauterine vertical transmission potential of COVID-19 infection in nine pregnant women: A retrospective review of medical records. Lancet.

[51] Nie R., Wang S., Yang O., Fan C., Liu Y., He W., Jiang M., Liu C., Zeng W., Wu J. (2020). Clinical features and the maternal and neonatal outcomes of pregnant women with coronavirus disease. MedRxiv.

[52] Baud D., Greub G., Favre G., Gengler C., Jaton K., Dubruc E., Pomar L. (2020). Second-Trimester Miscarriage in a Pregnant Woman With SARS-CoV-2 Infection. JAMA.

[53] Dong L., Tian J., He S., Zhu C., Wang J., Liu C., Yang J. (2020). Possible Vertical Transmission of SARS-CoV-2 From an Infected Mother to Her Newborn. JAMA.

[54] Fan C., Lei D., Fang C., Li C., Wang M., Liu Y., Bao Y., Sun Y., Huang J., Guo Y. (2020). Perinatal Transmission of 2019 Coronavirus Disease–Associated Severe Acute Respiratory Syndrome Coronavirus 2: Should We Worry?. Clin. Infect. Dis..

[55] Li Y., Zhao R., Zheng S., Chen X., Wang J., Sheng X., Zhou J., Cai H., Fang Q., Yu F. (2020). Lack of Vertical Transmission of Severe Acute Respiratory Syndrome Coronavirus 2, China. Emerg. Infect. Dis..

[56] Wang X., Zhou Z., Zhang J., Zhu F., Tang Y., Shen X. (2020). A Case of 2019 Novel Coronavirus in a Pregnant Woman with Preterm Delivery. Clin. Infect. Dis..

[57] Yin M., Zhang L., Deng G., Han C., Shen M., Sun H., Zeng F., Zhang W., Chen L., Luo Q. (2020). Severe acute respirato-ry syndrome coronavirus 2 (SARS-CoV-2) infection during pregnancy in China: A retrospective cohort study (Preprint). MedRxiv.

[58] Yu N., Li W., Kang Q., Xiong Z., Wang S., Lin X., Liu Y., Xiao J., Liu H., Deng D. (2020). Clinical features and obstetric and neonatal outcomes of pregnant patients with COVID-19 in Wuhan, China: A retrospective, single-centre, descriptive study. Lancet Infect. Dis..

[59] Zambrano L.I., Barahona F.I.C., Torres B.D.A., Bustillo C., Gonzales G., Chinchilla V.G., Martínez S.F.E., Reconco V.J.A., Sierra M., Aldana B.D.K. (2020). A pregnant woman with COVID-19 in Central America. Travel Med. Infect. Dis..

[60] Zeng H., Xu C., Fan J., Tang Y., Deng Q., Zhang W., Long X. (2020). Antibodies in Infants Born to Mothers with COVID-19 Pneumonia. JAMA.

[61] Zhu H., Wang L., Fang C., Peng S., Zhang L., Chang G., Xia S., Zhou W. (2020). Clinical analysis of 10 neonates born to mothers with 2019-nCoV pneumonia. Transl. Pediatr..

[62] Di Guardo F., Di Grazia F.M., Di Gregorio L.M., Zambrotta E., Carrara G., Gulino F.A., Tuscano A., Palumbo M. (2021). Poor maternal–neonatal outcomes in pregnant patients with confirmed SARS-Cov-2 infection: Analysis of 145 cases. Arch. Gynecol. Obstet..

[63] Badr D.A., Mattern J., Carlin A., Cordier A.G., Maillart E., El Hachem L., El Kenz H., Andronikof M., De Bels D., Damoisel C. (2020). Are clinical outcomes worse for pregnant women at ≥20 weeks’ gestation infected with coronavirus disease 2019? A multicenter case-control study with propensity score matching. Am. J. Obstet. Gynecol..

[64] Allotey J., Stallings E., Bonet M., Yap M., Chatterjee S., Kew T., Debenham L., Llavall A.C., Dixit A., Zhou D. (2020). Clinical manifestations, risk factors, and maternal and perinatal outcomes of coronavirus disease 2019 in pregnancy: Living systematic review and meta-analysis. BMJ.

[65] Burwick R.M., Yawetz S., Stephenson K.E., Collier A.R.Y., Sen P., Blackburn B.G., Kojic E.M., Hirshberg A., Suarez J.F., Sobieszczyk M.E. (2020). Compassionate Use of Remdesivir in Pregnant Women with Severe Coronavirus Disease. Clin. Infect. Dis..

[66] Wang K., Kang S., Tian R., Zhang X., Wang Y. (2020). Imaging manifestations and diagnostic value of chest CT of coronavirus disease 2019 (COVID-19) in the Xiaogan area. Clin. Radiol..

[67] Hu Z., Song C., Xu C., Jin G., Chen Y., Xu X., Ma H., Chen W., Lin Y., Zheng Y. (2020). Clinical characteristics of 24 asymptomatic infections with COVID-19 screened among close contacts in Nanjing, China. Sci. China Life Sci..

[68] Wang Y., Liu Y., Liu L., Wang X., Luo N., Li L. (2020). Clinical Outcomes in 55 Patients with Severe Acute Respiratory Syndrome Coronavirus 2 Who Were Asymptomatic at Hospital Admission in Shenzhen, China. J. Infect. Dis..

[69] Inchingolo R., Smargiassi A., Moro F., Buonsenso D., Salvi S., Del Giacomo P., Scoppettuolo G., Demi L., Soldati G., Testa A.C. (2020). The diagnosis of pneumonia in a pregnant woman with coronavirus disease 2019 using maternal lung ultrasound. Am. J. Obstet. Gynecol..

[70] Poon L.C., Abramowicz J.S., Asta D.A., Sande R., Haar G., Maršal K., Brezinka C., Miloro P., Basseal J., Westerway S.C. (2020). ISUOG Safety Committee Position Statement on safe performance of obstetric and gynecological scans and equipment cleaning in context of COVID. Ultrasound Obstet. Gynecol..

[71] Nouvenne A., Zani M.D., Milanese G., Parise A., Baciarello M., Bignami E.G., Odone A., Sverzellati N., Meschi T., Ticinesi A. (2020). Lung Ultrasound in COVID-19 Pneumonia: Correlations with Chest CT on Hospital admission. Respiration.

[72] Falgarone G., Pamoukdjian F., Cailhol J., Auregan G.A., Guis S., Bousquet G., Bouchaud O., Seror O. (2020). Lung ultrasound is a reliable diagnostic technique to predict abnormal CT chest scan and to detect oxygen requirements in COVID-19 pneumonia. Aging.

[73] Yassa M., Birol P., Mutlu A.M., Tekin A.B., Sandal K., Tug N. (2021). Lung Ultrasound Can Influence the Clinical Treatment of Pregnant Women with COVID. J. Ultrasound Med..

[74] Maraschini A., Corsi E., Salvatore M.A., Donati S. (2020). ItOSS COVID-19 Working Group. Coronavirus and birth in Italy: Results of a national population-based cohort study. Ann. Ist. Super. Sanita.

[75] Della Gatta A.N., Rizzo R., Pilu G., Simonazzi G. (2020). Coronavirus disease 2019 during pregnancy: A systematic review of reported cases. Am. J. Obstet. Gynecol..

[76] Knight M., Bunch K., Vousden N., Morris E., Simpson N., Gale C., Brien O.P., Quigley M., Brocklehurst P., Kurinczuk J.J. (2020). Characteristics and outcomes of pregnant women admitted to hospital with confirmed SARS-CoV-2 infection in UK: National population based cohort study. BMJ.

[77] World Health Organization Collaborating Centre for Women’s Health University of Birmingham COVID-19 in Pregnancy (PregCOV-19 LRS). www.birmingham.ac.uk/research/whocollaborating-centre/pregcov/index.aspx.

[78] Oncel M.Y., Akın I.M., Kanburoglu M.K., Tayman C., Coskun S., Narter F., Er I., Oncan T.G., Memisoglu A., Centinkaya M. (2021). A multicenter study on epidemiological and clinical characteristics of 125 newborns born to women infected with COVID-19 by Turkish Neonatal Society. Eur. J. Pediatr..

[79] Yang Z., Liu Y. (2020). Vertical Transmission of Severe Acute Respiratory Syndrome Coronavirus 2: A Systematic Review. Am. J. Perinatol..

[80] Chen M.M., Coakley F.V., Kaimal A., Laros R.K. (2008). Guidelines for Computed Tomography and Magnetic Resonance Imaging Use During Pregnancy and Lactation. Obstet. Gynecol..

[81] Youssef A., Serra C., Pilu G. (2020). Lung ultrasound in the coronavirus disease 2019 pandemic: A practical guide for obstetricians and gynecologists. Am. J. Obstet. Gynecol..

[82] Demi L., Demi M., Smargiassi A., Inchingolo R., Faita F., Soldati G., Task Force Group (2014). Ultrasonography in lung pathologies: New perspectives. Multidiscip. Respir. Med..

[83] Smargiassi A., Inchingolo R., Soldati G., Copetti R., Marchetti G., Zanforlin A., Giannuzzi R., Testa A., Nardini S., Valente S. (2013). The role of chest ultrasonography in the management of respiratory diseases: Document II. Multidiscip. Respir. Med..

[84] Soldati G., Demi M., Smargiassi A., Inchingolo R., Demi L. (2019). The role of ultrasound lung artifacts in the diagnosis of respiratory diseases. Expert Rev. Respir. Med..

[85] Schnettler W.T., Al Ahwel Y., Suhag A. (2020). Severe acute respiratory distress syndrome in coronavirus disease 2019–infected pregnancy: Obstetric and intensive care considerations. Am. J. Obstet. Gynecol. MFM.

[86] Soldati G., Smargiassi A., Inchingolo R., Buonsenso D., Perrone T., Briganti D.F., Perlini S., Torri E., Mariani A., Mossolani E.E. (2020). Is There a Role for Lung Ultrasound During the COVID -19 Pandemic?. J. Ultrasound Med..

[87] Buonsenso D., Piano A., Raffaelli F., Bonadia N., de Gaetano Donati K., Franceschi F. (2020). Point-of-Care Lung Ultrasound find-ings in novel coronavirus disease-19 pneumoniae: A case report and potential applications during COVID-19 outbreak. Eur. Rev. Med. Pharmacol. Sci..

[88] Kalafat E., Yaprak E., Cinar G., Varli B., Ozisik S., Uzun C., Azap A., Koc A. (2020). Lung ultrasound and computed tomographic findings in pregnant woman with COVID. Ultrasound Obstet. Gynecol..

